# Surface-Enhanced Raman Spectroscopy and Artificial Neural Networks for Detection of MXene Flakes’ Surface Terminations

**DOI:** 10.1021/acs.jpcc.4c01273

**Published:** 2024-04-10

**Authors:** Andrii Trelin, Anastasiia Skvortsova, Anastasia Olshtrem, Sergii Chertopalov, David Mares, Ladislav Lapcak, Martin Vondracek, Petr Sajdl, Vitezslav Jerabek, Jaroslav Maixner, Jan Lancok, Zdenek Sofer, Jakub Regner, Zdenka Kolska, Vaclav Svorcik, Oleksiy Lyutakov

**Affiliations:** † Department of Solid State Engineering, University of Chemistry and Technology, Prague 16628, Czech Republic; ‡ Institute of Physics of the Czech Academy of Sciences, Prague 18220, Czech Republic; § Department of Microelectronics, Faculty of Electrical Engineering, Czech Technical University, Prague 16627, Czech Republic; ∥ Central Laboratories, University of Chemistry and Technology, Prague 16628, Czech Republic; ⊥ Department of Power Engineering, University of Chemistry and Technology, Prague 16628, Czech Republic; # Department of Inorganic Chemistry, University of Chemistry and Technology, Prague 16628, Czech Republic; ¶ Centre for Nanomaterials and Biotechnology, J. E. Purkyne University, Usti nad Labem 40096, Czech Republic

## Abstract

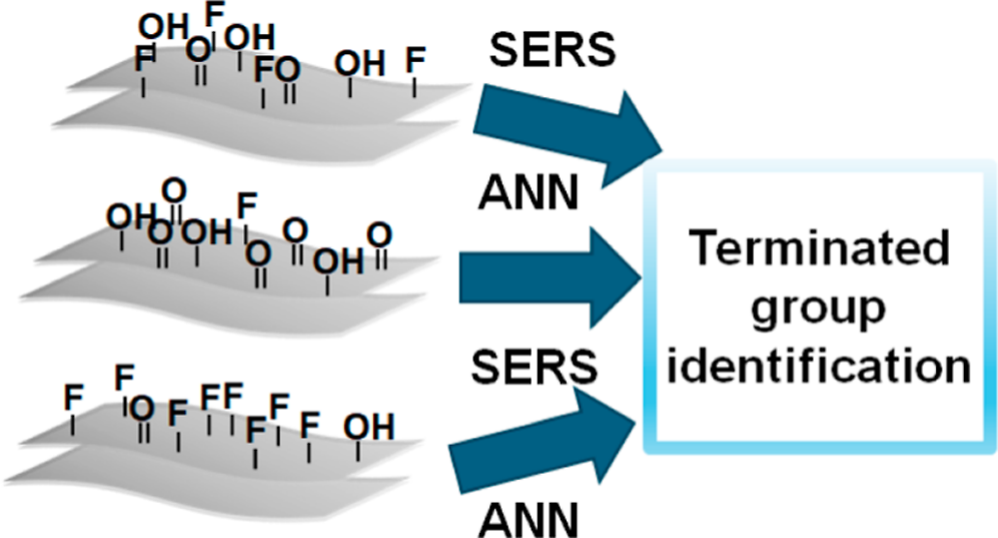

The properties of MXene flakes, a new class of two-dimensional materials, are strictly determined by their surface termination. The most common termination groups are oxygen-containing (=O or –OH) and fluorine (−F), and their relative ratio is closely related to flake stability and catalytic activity. The surface termination can vary significantly among MXene flakes depending on the preparation route and is commonly determined after flake preparation by using X-ray photoelectron spectroscopy (XPS). In this paper, as an alternative approach, we propose the combination of surface-enhanced Raman spectroscopy (SERS) and artificial neural networks (ANN) for the precise and reliable determination of MXene flakes’ (Ti_3_C_2_T_*x*_) surface chemistry. Ti_3_C_2_T_*x*_ flakes were independently prepared by three scientific groups and subsequently measured using three different Raman spectrometers, employing resonant excitation wavelengths. Manual analysis of the SERS spectra did not enable accurate determination of the flake surface termination. However, the combined SERS-ANN approach allowed us to determine the surface termination with a high accuracy. The reliability of the method was verified by using a series of independently prepared samples. We also paid special attention to how the results of the SERS-ANN method are affected by the flake stability and differences in the conditions of flake preparation and Raman measurements. This way, we have developed a universal technique that is independent of the above-mentioned parameters, providing the results with accuracy similar to XPS, but enhanced in terms of analysis time and simplicity.

## Introduction

In the nanomaterials science community, one of the very popular trends is closely related to the development and utilization of two-dimensional (2D) materials.^1^ Starting from the graphene era, several classes of 2D materials, including metal oxides, single element(s) borophene, or phosphorene as well as 2D transition-metal dichalcogenides, can be mentioned.^2−5^ One of the more recent ones, which received considerable scientific attention, was discovered by Gogotsi and Barsoum in 2011 and designated by them as MXenes.^6,7^ It is not surprising since the MXenes benefit from a range of advanced properties related to their 2D structure and high concentration of mobile charge carriers.^8−10^ In addition, MXenes have an abundant number of catalytically active centers on their surface (including both edges and basal planes), which ensure their potential utilization in contemporary energy conversion and storage fields.^11,12^

The general formula of MXenes is M_*n*+1_X_*n*_T, where M is an early transition metal, X is carbon and/or nitrogen, and T_*x*_ is the terminated group (commonly –F, –OH, or =O). While the “basic” properties of MXenes are primarily determined by the transition-metal atoms (as well as carbon or nitrogen monolayer within), the presence and number of surface termination groups determine such properties of MXenes as their redox activity, stability, and surface charge.^13,14^ The structure of the inner MXene layers is strongly and consistently determined by the MAX phase used for flakes’ preparation. However, the composition and number of termination groups are rather dependent on the specific flakes’ preparation method and can vary significantly with slight variations in the etching and delamination process.^15−17^ Consequently, the properties of MXenes, as well as their range of applications, can vary significantly within the framework of the same M_*n*+1_X_*n*_ structure owing to the uncertainty in the relative ratio of surface termination groups (i.e., “T”). To illustrate, oxygen-containing surface termination groups have demonstrated higher catalytic activity, whereas fluorine surface termination can enhance flakes’ stability.^18−21^

The surface termination of MXene flakes plays a crucial role in determining their stability and functionality, making it an important parameter. Therefore, there is a high demand for accurate, efficient, and user-friendly methods to characterize the surface of the MXene flakes. The X-ray photoelectron spectroscopy (XPS) technique is commonly used to determine the surface termination of flakes with high precision and accuracy.^22,23^ However, this technique requires specialized equipment, trained operators, long measurement times, and manual data evaluation. As an alternative, Raman spectroscopy (or surface-enhanced Raman spectroscopy—SERS) can be utilized to determine the surface termination of MXene flakes.^24^ However, the Raman and SERS spectra of MXenes often exhibit complex patterns, making precise determination of the flakes’ surface chemistry challenging. Nevertheless, the application of machine learning algorithms allows for the evaluation of such complex SERS spectra.^25−27^ It has been demonstrated that these algorithms can extract accurate and quantitative information even from highly intricate and overlapping spectral patterns.^28−30^

In this work, we propose for the first time the utilization of the SERS-artificial neural networks (ANN) approach to determine the surface termination of MXene flakes. As a model system, we focused on Ti_3_C_2_T_*x*_ flakes, which were independently prepared in three different laboratories. Additionally, we employed three Raman spectrometers to collect a comprehensive spectral database and validate the results obtained by using the SERS-ANN method. The outcome of this research is a straightforward and accurate approach for characterizing the surface termination of flakes, which can be executed within a few seconds and does not require additional operator learning or training.

## Experimental Section

A detailed description of the used materials, sample preparation, and characterization methods is given in the Supporting Information. In this work, we used three kinds of MXenes (in particular—commonly used MXenes—Ti_3_C_2_T_*x*_) prepared using the etching/delamination of self-made or commercially supplied MAX phase with optional utilization of ultrasound-assisted flakes’ delamination. The structure of created flakes was verified using X-ray diffraction (XRD) and transmission electron microscopy (TEM) measurements. Subsequently, the surface termination of flakes was determined by XPS, and the obtained data were used for ANN training or SERS-ANN results’ verification. SERS spectra were collected on three Raman spectrometers, with utilization of 785 nm excitation wavelengths. The results of SERS-ANN-based determination of flakes’ termination were checked on separately prepared samples, previously unknown for ANN.

## Results and Discussion

### Main Experimental Concept

**Figure 1:**
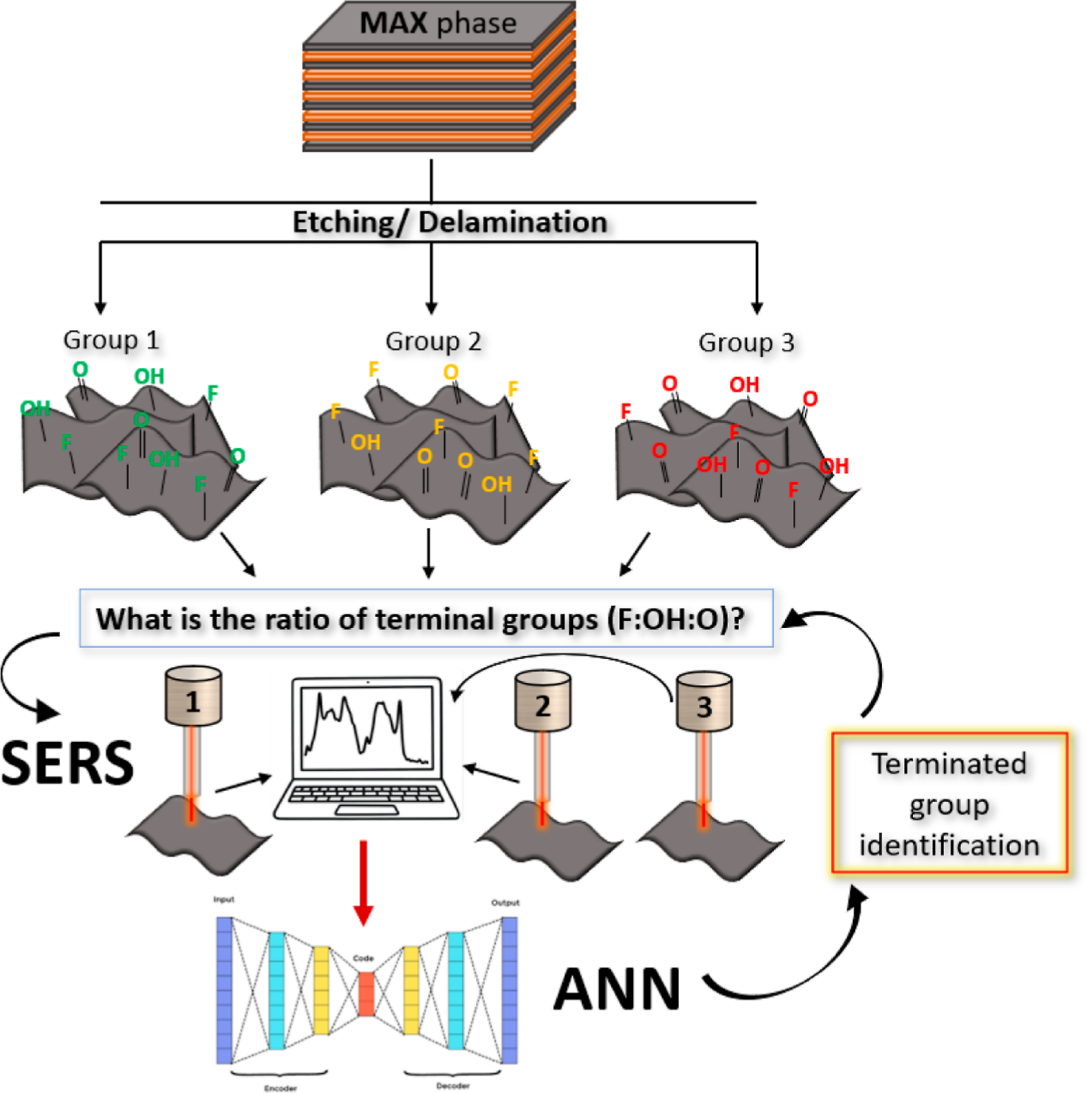
Schematic representation of the proposed experimental concept.

Our main experimental concept is illustrated in Figure 1. Initially, a variety of Ti_3_C_2_T_*x*_ flakes were prepared independently by four scientific teams, each having previous experience with MAX phase etching and delamination. The flakes’ production was carried out without communication between the operators (five operators were totally involved). Subsequently, the Ti_3_C_2_T_*x*_ flakes were drop-cast onto the Si substrate, dried, and subjected to Raman measurements (in particular, SERS measurements). We utilized a Raman excitation wavelength of 785 nm, which corresponds to the plasmon absorption band of the flakes. To ensure the reliability and robustness of the SERS-ANN-based determination of flakes’ surface termination, we performed the SERS measurements on three different spectrometers, involving several operators who worked independently. The resulting spectral database consisted of approximately 130,000 spectra, which were subsequently used for ANN training and validation. During ANN training, we introduced variability by excluding spectra obtained from a specific spectrometer or from one of the research groups. As a control method, we employed XPS for unambiguous determination of the flakes’ surface composition. While both =O and –OH groups in the oxygen termination exhibit similar functionality (both being potentially redox-active or oxidative-unstable sites), we primarily focused on identifying the fluorine termination (as a competitor to oxygen termination). Specifically, we utilized the Ti/F ratio as an indicator of the presence of fluorine groups or the absence of oxygen-containing termination.

### Characterization of Ti_3_C_2_T_*x*_ Flakes

**Figure 2:**
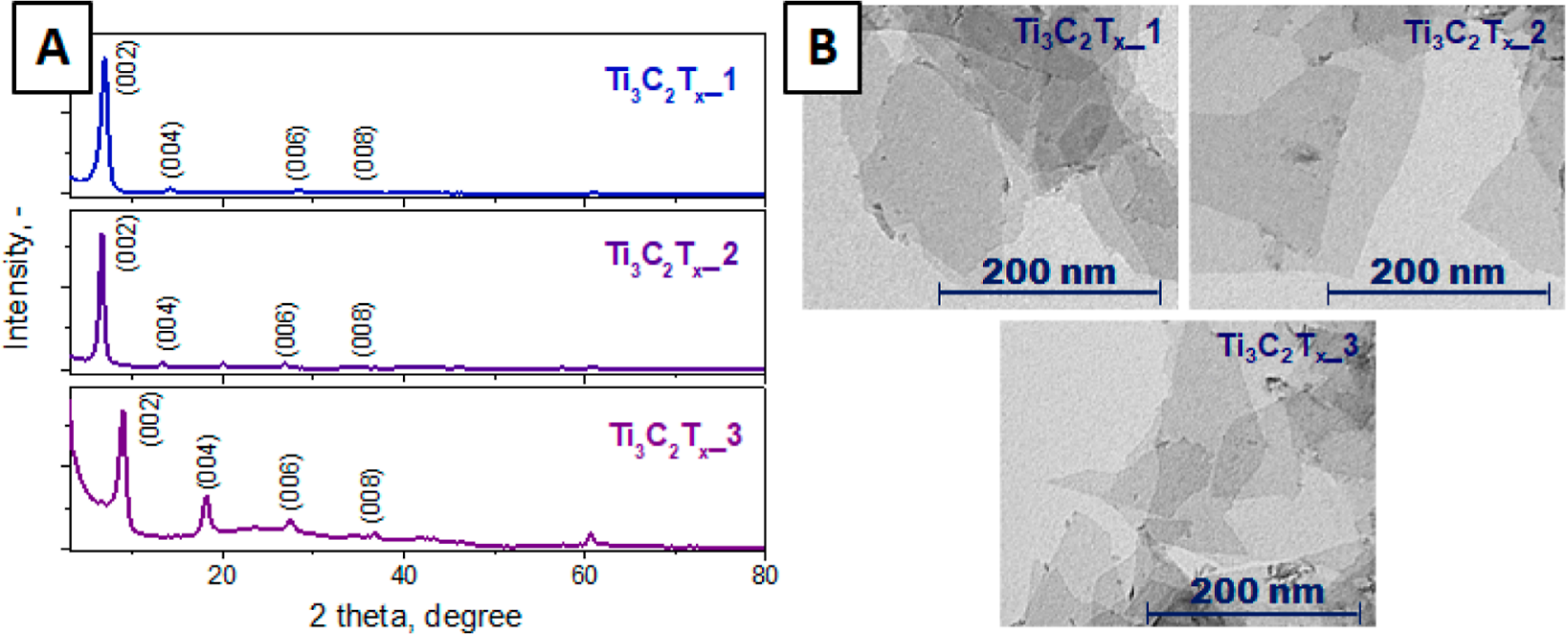
Typical XRD patterns (A) and TEM images (B) of MXene flakes prepared by different scientific teams.

The 2D structure of Ti_3_C_2_T_*x*_ flakes was confirmed through XRD and TEM measurements. The typical results obtained are listed in Figure 2. The XRD patterns exhibit characteristic reflexes from (004), (006), and (008) planes, which align with previously reported observations. Notably, the more prominent low-angle (002) peak confirms the 2D nature of the flakes, while the absence of the peak at 39.3° indicates the complete etching of the aluminum (Al) layers. The variation in the relative peaks’ intensity can be attributed to the different experimental pathways of flakes’ preparation. Moreover, the shifting of the (002) peak position indicates variations in the interflake distance, implying different levels of dense packing among the flakes. The 2D nature of the flakes is further substantiated by TEM measurements, as shown in Figure 2B. The TEM image exhibits fully etched and delaminated flakes without the presence of accordion-like structures and with minimal defect content across samples from all participating scientific groups. The lateral sizes of the flakes ranged from 100 nm to 1 μm, and the fluctuations in size can be attributed to the distinct methods employed by each group for the preparation of the 2D material (for example, “MILD” or “sonic” preparation routes as well as previous NaOH etching, followed by LiF/HCl etching, as outlined in Table 1 and experimental details in the Supporting Information).

**Table 1:** A Number of Control (Previously Unknown for SERS-ANN) Ti_3_C_2_T_*x*_ Samples and Measured Spectra for Verification of the SERS-ANN Approach

samples description	number of Ti_3_C_2_T_*x*_ samples	number of spectra, Raman1/Raman2/Raman3
***Group 1***, laboratory-prepared MAX phase, MILD and/or ultrasonic etching/delamination	11	1303/1240/1355
***Group 2***, commercially supplied MAX phase, MILD and/or ultrasonic etching/delamination	10	1214/1425/1297
***Group 3***, laboratory-prepared MAX phase, MILD etching, in some cases–postpreparative addition ultrasonication	7	861/817/843
***Group 4***, commercially supplied MAX phase, previous etching in NaOH for various times, final MILD etching	8	937/935/1241

**Figure 3:**
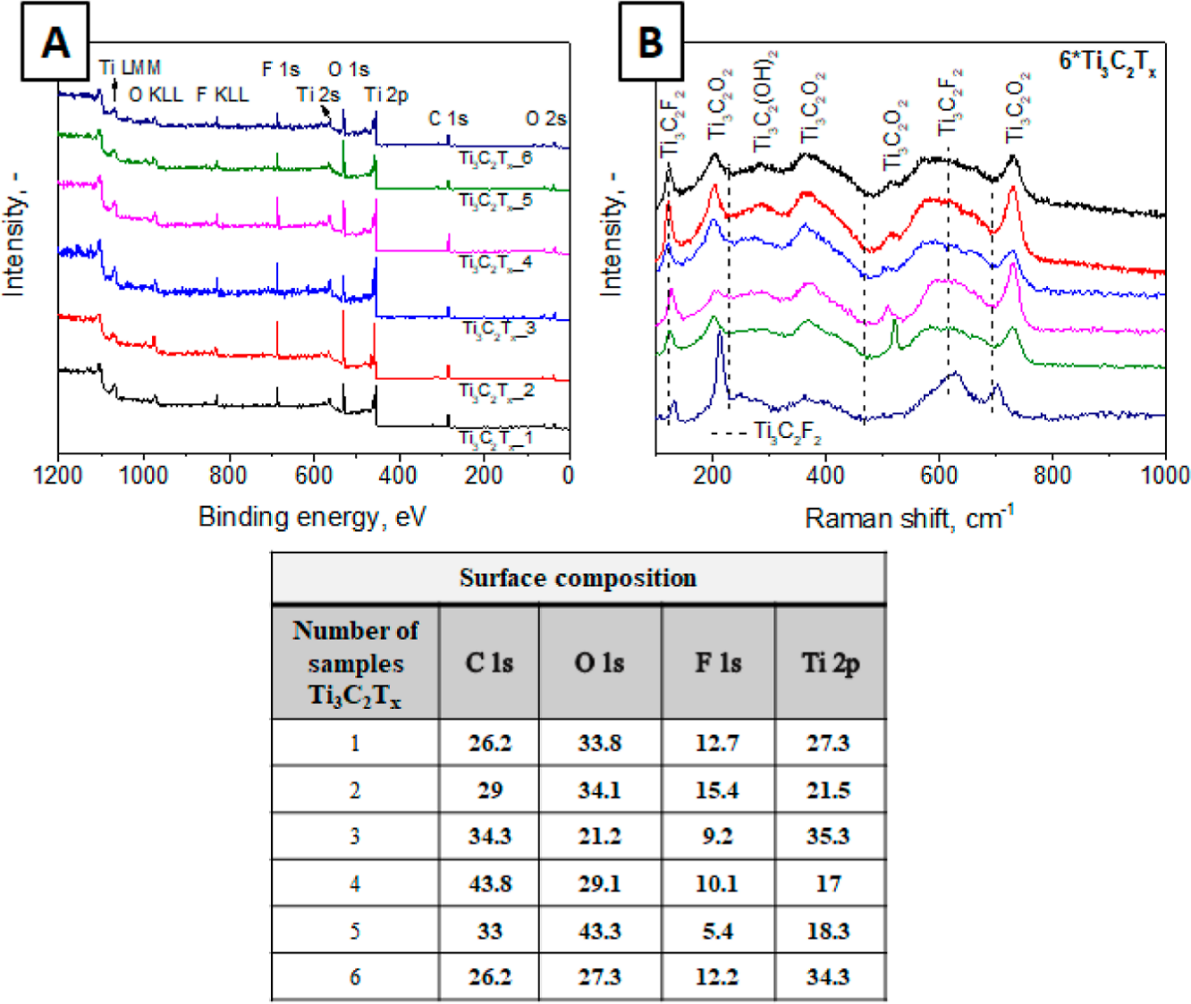
Examples of typical XPS raw spectra (A) and SERS spectra (B) measured on randomly chosen Ti_3_C_2_T_*x*_ flakes prepared by different teams. The table shows the element composition on the flakes’ surface (in at. %), calculated from XPS data.

After confirming the 2D structure of the material, we proceeded with the characterization of the flakes’ surface chemistry, first performed by XPS and subsequently by SERS-ANN techniques. Each Ti_3_C_2_T_*x*_ sample underwent XPS measurements, which were conducted once for each specific sample, including those used for SERS-ANN training and control samples. The typical survey XPS spectra are presented in Figure 3A, and the corresponding XPS-determined surface composition of the flakes is shown in the table in Figure 3. The XPS results reveal significant variations in the surface termination of the flakes. These variations are evident from the diverse composition observed, indicating differences in the surface chemistry of the flakes, appearing as a result of different routes of flakes’ preparation as well as previous etching of the MAX phase in NaOH. To optimize the SERS-ANN approach, we employed surface fluorination (specifically, the Ti/F ratio, to avoid possible mistakes due to carbon contamination, see Figure S1 or TiO_2_ formation) as a marker for the determination of the flakes’ surface termination. The XPS results regarding the surface composition of the flakes demonstrate that the Ti/F ratio varies within the range of 1.3 to 5 (corresponded to flakes etched in HF, where the amounts of −F termination is large and flakes previously etched in NaOH, where the oxygen termination is dominant). Such variations in surface chemistry indicate that the flakes will exhibit different functionalities and stabilities. This underscores the importance of characterizing the surface termination of the flakes after preparation as it directly impacts their properties and behavior.

### SERS-ANN Approach Verification

Figure 3B presents several examples of SERS spectra measured on different groups of flakes. The typical SERS response of flakes is a superposition of four active Raman modes: *E*_g_ and *A*_1g_ in-plane and out-of-plane vibrations of Ti and C atoms, with some contribution from surface-terminated groups. The prominent peak at 122 cm^–1^ arises from the coupling of the excitation laser wavelength to MXene plasmonic modes.^31^ The 150–270 cm^–1^ spectral region can be attributed to in-plane and out-of-plane vibrations of titanium and carbon atoms as well as the vibration of surface termination groups. The 230–470 cm^–1^ spectral region corresponds to the vibrations of surface groups attached to titanium atoms (so-called T_*x*_ region). This region was proposed for the determination of flakes’ surface termination earlier.^24^ The last 580 and 730 cm^–1^ regions can be assigned dominantly to carbon vibrations. It is important to note that variations in the surface termination of MXene flakes will not solely affect the spectra in the T_*x*_ region but also the *E*_g_ and *A*_1g_ vibration modes, as previously reported.^32^ In fact, all spectral patterns can be affected by changes in the flake surface termination. The SERS spectra exhibit complex patterns with overlapping peaks, making a direct, precise, and accurate determination of the flakes’ surface termination by manual analysis challenging. However, this challenge can be overcome by utilizing ANN, which have the capability to reveal and interpret hidden features within complex spectral patterns.^33−37^

### SERS-ANN Determination of Ti_3_C_2_T_*x*_ Flakes’ Surface Termination—An Ideal Case

**Figure 4:**
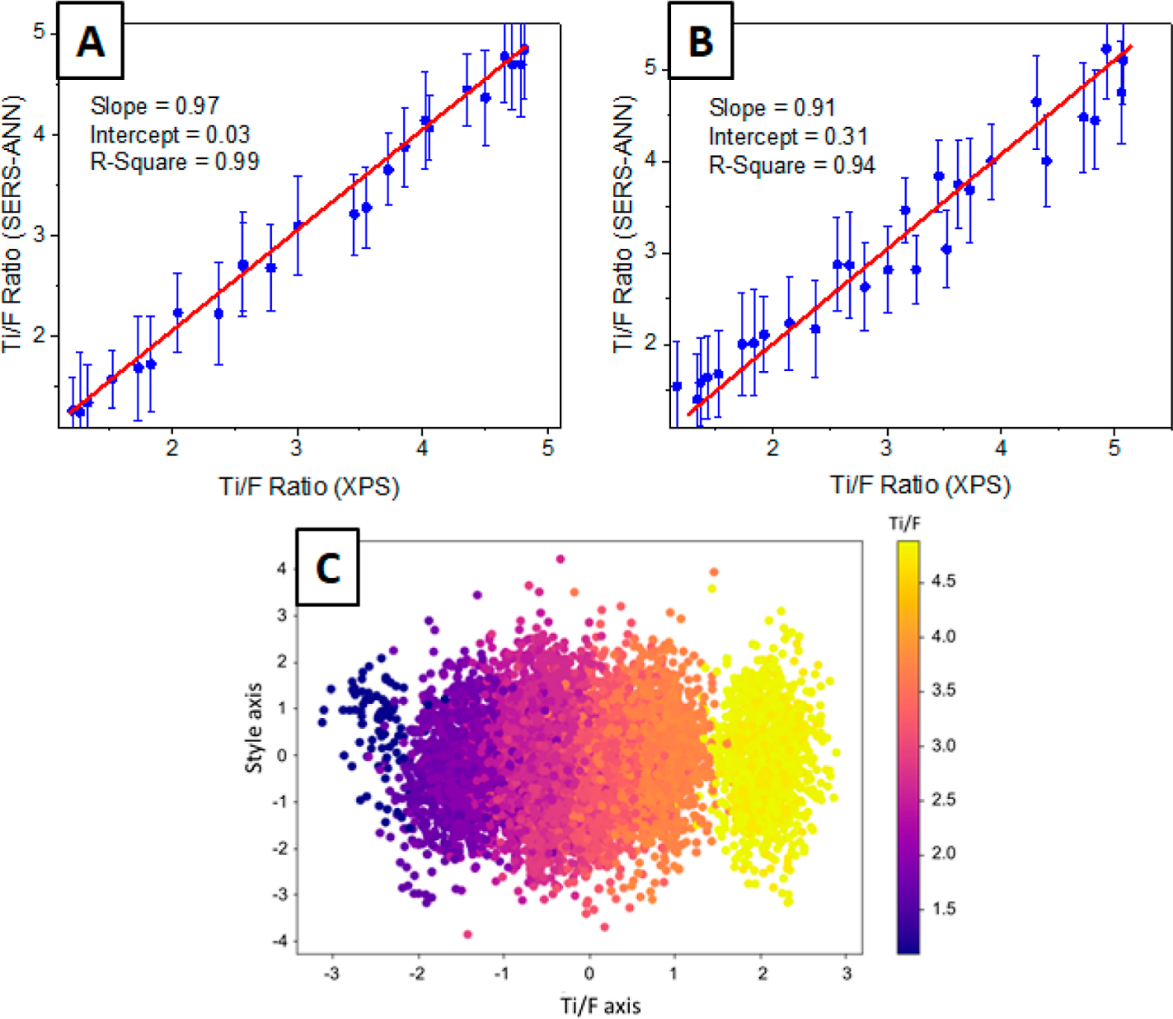
SERS-ANN-based determination of flakes’ surface termination (represented by C/F ratio, which determines the degree of flakes’ surface fluorination, opposite to the amount of oxygen-containing termination): (A) representation of an “ideal” case, where the part of Ti_3_C_2_T_*x*_ flakes (prepared by one partner) were excluded from the ANN learning and SERS-ANN verification. (B) Results of all flakes’ analysis (including flakes that tended to degrade, see text). (C) Projection of SERS-ANN results in 2D space, where the *X*-axis corresponds to top flakes’ surface termination, while the *Y*-axis represents the complex parameters (deviation in spectra due to various preparation and measurements routes).

For ANN learning, a database consisting of approximately 130,000 spectra was utilized (the details of the ANN structure and learning process can be found in the Supporting Information). Subsequently, the SERS-ANN approach was implemented to predict the surface termination of MXene flakes that were previously unknown to the ANN (Table 1). The main results are presented in Figure 4A,B, as well as Figure S2 (provided in the Supporting Information). It should be noted that a portion of the spectra was excluded during the interpretation process, as described in the “Raman spectrometer impact” section. Figure 4A demonstrates the predicted surface termination of the flakes (determined by SERS-ANN) compared to the actual surface termination (determined by XPS) in an “ideal” scenario, where certain data were excluded from the analysis (more details are provided below). As evident from the figure, there is an excellent correlation between the predicted and actual surface termination with a slope coefficient of 0.97 (ideally, it should be 1) and an R-square value 0.99. This close-to-ideal correlation between XPS and SERS-ANN results was achieved by utilizing flakes from three different groups (generally, four groups were involved) and spectra measured on two higher scientific grade Raman spectrometers. Therefore, it can be concluded that in some “ideal” cases, such as using high-quality flakes and higher scientific grade Raman spectrometers, the implementation of the SERS-ANN approach provides information about the surface termination of MXene flakes with high precision and accuracy.

### SERS-ANN Determination of Ti_3_C_2_T_*x*_ Flakes’ Surface Termination—The Impact of Flakes’ Quality

However, when the flakes prepared by the fourth group were included, there was a slight decrease in the accuracy of the SERS-ANN detection of the flakes’ surface termination. In this case, the slope value was 0.91, and the R-square value decreased to 0.94, indicating a slightly less accurate but still acceptable determination of the flakes’ surface composition. The drop in accuracy was somewhat surprising, as the XRD and TEM analyses (see previous Figures 2 and 3) did not show any significant differences between the flakes prepared by different groups and operators. However, more detailed online SERS measurements revealed that the flakes from the third group had a tendency to degrade during the measurement. Figure S3 illustrates several spectra measured at one point over a relatively short time interval, showing an apparent change in the spectral pattern between the measurements. At the moment, we cannot definitively identify the exact cause of the flakes’ degradation as the investigation of flakes’ degradation is not the primary focus of our work. Since the SERS measurements of flakes from all groups were carried out under the same conditions, the degradation will likely be associated with a large number of defects in the flakes’ inner structure (Ti or C vacancies) that appeared either during the preparation of the MAX phase or during its etching/delamination.^38,39^ It is also important to note that despite the degradation of the flakes, the SERS-ANN approach still enabled the determination of the surface termination in a relatively accurate manner (Figure 4B). Therefore, we can conclude that in our SERS-ANN approach, while the quality and stability of the flakes’ internal structure are desirable parameters, they are not strictly mandatory for determining the surface termination of the flakes.

### SERS-ANN Determination of Ti_3_C_2_T_*x*_ Flakes’ Surface Termination—Impact of the Raman Spectrometer

Another important factor to consider is the quality of the Raman spectrometer used for collecting SERS spectra. As mentioned earlier, we employed two high-quality scientific-grade Raman spectrometers and a third, more portable device. The results presented in Figure 4A,B were obtained using the first two, both for training the ANN and verifying the SERS-ANN method on “unknown” samples. The results of SERS-ANN surface termination detection, which include data from the third (portable) spectrometer, are provided in Figure S2. In Figure S4, the SERS spectra measured with all three spectrometers are shown, and no fundamental differences between them are observed. However, when the SERS-ANN results obtained using the third spectrometer were compared to those from the previous case (Figures S2 vs 4), a significant decrease in accuracy was observed. The determination of the Ti/F ratio was less accurate, and there were instances where the SERS-ANN results and XPS control data were completely uncorrelated.

Based on the data presented, we can draw the following conclusions: (i)—the SERS-ANN method is suitable for determining the surface termination of flakes, primarily when using higher scientific-grade SERS spectrometers; (ii)—in this case, the SERS-ANN approach is insensitive to the spectrometer (as long as it is properly calibrated), and the data can be transferred from one instrument to another; (iii)—the SERS-ANN approach may not yield accurate results when using instruments of lower quality (such as small portable Raman devices), which can be classified as having a lower scientific class.

### Impact of Sample Preparation and SERS Measurement Style

Lastly, we took into account the concept of “style” during the utilization of the SERS-ANN approach. This concept encompassed a wide range of parameters, including differences in sample preparation and SERS measurements as well as potential variations in other “unknown” parameters. The proposed ANN design allowed us to separate the difference in spectra attributed to the “style” from those related to the flakes’ surface termination. The results of this spectral interpretation are presented in Figure 4C, where each individual point corresponds to one spectrum measured on control Ti_3_C_2_T_*x*_ samples. The *X*-axis characterizes the surface termination (in terms of Ti/F ratio), while the *Y*-axis reveals the spectrum position according to the style. It is evident that samples with similar surface termination can differ significantly in their style. At the same time, the projection of the samples position in 2D space on the *X*-axis (“responsible” for the Ti/F ration) is invariant in terms of surface termination and is independent of the “style”. In other words, the proposed SERS-ANN approach can be considered as universal and capable of revealing the surface termination of flakes independently of the flake preparation route and measurement conditions.

## Conclusions

In this study, we propose the utilization of the SERS-ANN approach for determining the surface termination of MXene flakes, focusing on Ti_3_C_2_T_*x*_ flakes that were independently prepared by three scientific teams. SERS measurements were performed using three Raman spectrometers of different qualities, and the resulting spectral database was used for ANN learning. The main objective of the ANN was to determine the degree of surface fluorination, assuming that a lower degree of surface termination corresponds to a higher content of oxygen-containing surface-terminated groups and vice versa. The results of the SERS-ANN approach were validated using a set of separately prepared Ti_3_C_2_T_*x*_ flakes, previously unknown for ANN. The obtained results clearly indicate that the SERS-ANN approach can be considered as a useful and reliable tool for the determination of the MXene surface termination. The best results were achieved when using more stable flakes without defects or measurement-induced degradation as well as high-quality scientific-grade Raman spectrometers. Although the utilization of less stable flakes for both SERS-ANN training and verification led to a slightly decreased accuracy, the results still demonstrated satisfactory reliability. However, when a lower scientific quality Raman spectrometer was used, there was a significant decrease in the accuracy of the SERS-ANN approach, leading to unreliable results on the control samples. Furthermore, we demonstrated that the SERS-ANN approach is applicable independent of the flakes’ preparation and measurement routes. It is important to note that the proposed SERS-ANN approach for determining the surface termination of flakes is favored for its simplicity and minimal requirements for sample measurements and staff training.

## Data Availability

Main data presented in this study are available at https://zenodo.org/records/10715735.

## Supporting Information Available

Ti_3_C_2_T_*x*_ flakes preparation and characterization and SERS measurements; “bad” results of the revealing of Ti_3_C_2_T_*x*_ flakes’ SERS-ANN-based surface termination; demonstration of Ti_3_C_2_T_*x*_ flakes’ degradation during the Raman measurements; comparison of SERS spectra measured with different Raman spectrometers; and ANN design and implementation (PDF)
