# Supplementary material for: Surface-Enhanced Raman Spectroscopy and Artificial Neural Networks for Detection of MXene Flakes’ Surface Terminations
Source: J Phys Chem C Nanomater Interfaces. 2024 Apr 10;128(16):6780–7. doi: 10.1021/acs.jpcc.4c01273 (PMC11056973; doi:10.1021/acs.jpcc.4c01273)
Supplement: Supplementary file 1 — jp4c01273_si_001.pdf [file jp4c01273_si_001.pdf]

Supporting Information for:

Surface Enhanced Raman Spectroscopy and Artificial Neural Networks for Detection of MXene Flakes Surface Terminations

Anastasia Olshtrem[a], Andrii Trelin[a], Sergii Chertopalov[b], Anastasiia Skvortsova[a], David Mares[c], Ladislav Lapcak[d], Martin Vondracek[b], Petr Sajdl[e], Vitezslav Jerabek[c], Jaroslav Maixner[d], Jan Lancok[b], Zdenek Sofer[f], Jakub Regner[f], Zdenka Kolska[g], Vaclav Svorcik[a], Oleksiy Lyutakov[a,*]

[a] *Department of Solid State Engineering, University of Chemistry and Technology, 16628 Prague, Czech Republic*

[b] *Institute of Physics of the Czech Academy of Sciences, Prague 18220, Czech Republic*

[c] *Department of Microelectronics, Faculty of Electrical Engineering, Czech Technical University, 166 27 Prague, Czech Republic*

[d] *Central Laboratories, University of Chemistry and Technology, 16628 Prague, Czech Republic*

[e] *Department of Power Engineering, University of Chemistry and Technology, 16628 Prague, Czech Republic*

[f] *Department of Inorganic Chemistry, University of Chemistry and Technology, 16628 Prague, Czech Republic*

[g] *Centre for Nanomaterials and Biotechnology, J. E. Purkyne University, 40096 Usti nad Labem, Czech Republic*

---

* *Corresponding author: lyutakoo@vscht.cz*

1. Experimental details

$Ti_3C_2T_x$ flakes preparation

Group 1

Preparation of Ti_3AlC_2 MAX phase. The Ti, Al, and graphite powders in a 3.00:1.10:1.88 molar ratio were mixed with zirconia balls (diameter 3 mm) by a ball milling machine at 60 rpm for 3 h in a plastic jar. The mixed powders were transferred to an alumina boat and heated in a tube furnace (4016T, Clasic CZ). Before synthesis, vacuum pumping was carried out twice to a pressure of 2 mbar, followed by a purge with argon. The MAX phase synthesis was accomplished by heating the mixture at 10°C/min to 1550°C in an argon atmosphere followed by 2 h holding period at 1550°C and cooling down to room temperature at 10°C/min.

Preparation of MXene flakes. $Ti_3C_2T_x$ was synthesized by selective etching of aluminum atomic layers in Ti_3AlC_2 by the MILD etching method. To synthesize MILD $Ti_3C_2T_x$, 300 mg of lithium fluoride (LiF) was added to 6 mL of 37% hydrochloric acid (HCl) in a 50 mL plastic centrifuge tube. Then, 300 mg of sieved Ti_3AlC_2 MAX phase powder (particle size below 45 µm) was added to this solution and stirred at 22°C for 24 h to completely etch the aluminumaluminum layers. After etching, the mixture was repeatedly washed with deionized water by centrifugation at 6000 rpm for 5 min and decantation of the acidic supernatant until a supernatant was obtained with a pH ≈ 6. Delamination of MXene flakes was performed by hand-shaking of the sample for 5 min (15 mL DI water in a 50 mL plastic test tube) followed by centrifugation at 3500 rpm for 1 h to precipitate and separate the non-delaminated MXene.

Group 2

Preparation of Ti_3AlC_2 MAX phase Ti_3AlC_2 MAX phase (Titanium aluminum carbide, powder, ≤100 µm particle size) was supplied from Sigma Aldrich.

Preparation of MXene flakes. MAX phase etching was performed in LiF/HCl solution at RT for 20 h. After preparation, the flakes were carefully washed with DI and subjected to additional ultrasonication ("mild", with utilization of ultrasonic bath or "sonic" with utilization of high power ultrasound). The resulting crushed flakes were then centrifuged at 3500 rpm for 30 min.

Group 3

Preparation of Ti_3AlC_2 MAX phase. Synthesis of Ti_3AlC_2 was performed from elements in a KBr melt. Elemental powders of Ti, Al, and graphite were mixed in a stoichiometric molar ratio of 3 : 1.2 : 2. KBr in 1 : 1 weight ratio was added to the resulting mixture. The 24 h mixing process was carried out in a multidirectional mixer (Turbula, WAB, Switzerland), using ethanol as a liquid medium and 5 mm diameter zirconia milling balls. The dried powder was uni-axially pressed in a steel die under a pressure of 200 MPa. The pressed powder was then subjected to further encapsulation with KBr and placed in an alumina crucible lined with KBr salt bed. The resulting mixture was heated at 1300°C for an hour with a heating rate of 5°C/min. Upon cooling, Ti_3AlC_2 was obtained by dissolving the reaction mixture in water and subsequent washing it with hot water to eliminate the residual KBr salt content. To prevent agglomeration, the Ti_3AlC_2 powder was then rinsed in ethanol, dried overnight at 70 °C in an oven, and sieved through a 63 µm sieve.

Preparation of MXene flakes. 2g of Ti_3AlC_2 was immersed in 100 mL 40 % HF solution (Sigma-Aldrich) and stirred continuously under ambient conditions for 7 days. After which, the

samples were separated by repeated centrifugation and redispersion in water and finally separated by suction filtration. The etched product was dried in a vacuum oven for 24 h at 50°C.

Group 4

Preparation of the Ti_3AlC_2 MAX phase. The Ti_3AlC_2 MAX phase (Titanium aluminum carbide powder, ≤100 μm particle size) was supplied from Sigma Aldrich.

Preparation of MXene flakes. 0.5g of Ti_3AlC_2 was immersed in 100 ml of concentrated NaOH solution and boiled at different times (4-20 h.) for MAX phase preliminary etching. Subsequently, the preetched MAX phase was carefully washed by several centrifugation and re-dispersion in DI cycles up to pH ≈ 7. The final etching was performed in LiF/HCl solution at RT for 8 h. After preparation, the flakes were carefully washed with DI.

$Ti_3C_2T_x$ flakes characterization

TEM images of $Ti_3C_2T_x$ flakes were obtained with a JEOL JEM-1010 transmission electron microscope with SIS MegaView III digital camera. X-ray diffraction measurements were carried out using the Empyrean, Malvern Panalytical diffractometer with Cu $K_{<\alpha>}$ radiation source in *2θ-θ* diffraction mode, the Bragg-Brentano geometry. X-ray photoelectron spectroscopy (XPS) was performed using an Omicron Nanotechnology ESCAProbeP spectrometer with a monochromated Al K Alpha X-ray source operating at 1486.6 eV.

SERS measurements

Raman spectrometer 1. Raman analysis was performed using the inVia™ Raman microscope on disperse Raman spectrometer (Renishaw) equipped with a confocal microscope Olympus and thermoelectrically cooled CCD detector. As an excitation source was used diode laser (wavelength 785 nm). Measurement conditions were 2 mW laser power, 5 s acquisition time per scan, and 10 repetitions. Spectra were recorded at a resolution of 2 cm^{-1} in the 3200-100 cm^{-1} wavenumber range.

Raman spectrometer 2. Raman spectra were measured on Nicolet Almega XR Raman spectrometer with a 785 nm excitation wavelength (Thermo Scientific, France). The spectra were measured 10 times, each of them with 30 s accumulation time. Measurement conditions were 2,35 mW laser power, 10 s acquisition time per scan, and 1 repetition. Spectra were recorded at a resolution of 2 cm^{-1} in the 3200-100 cm^{-1} wavenumber range.

Raman spectrometer 3. Raman spectra were measured on a portable ProRaman-L spectrometer with 785 nm excitation wavelengths. Measurement conditions were 15 mW laser power, 30 s accumulation time, and 10 repetitions. Spectral maps were analyzed in 1 area of every sample

(~300 spectra total); each area was approximately 500 × 500 µm^2 with a step of 60 µm between measurement spots. Spectra were recorded at a resolution of 2 cm^{-1} in the 3000-150 cm^{-1} wavenumber range.

2. Additional experimental results

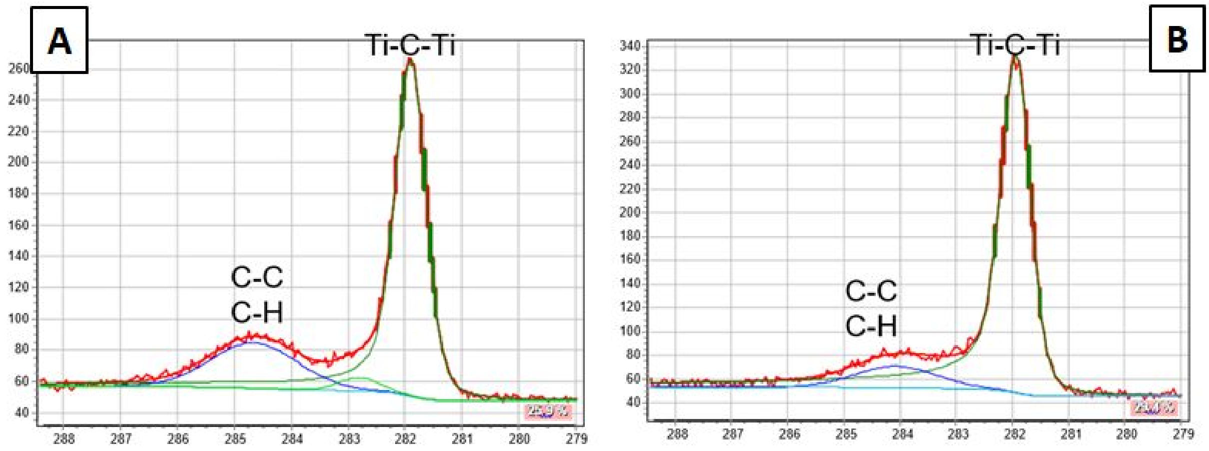

Figure S1 Deconvolution of XPS carbon peak, revealing the presence of both, graphitic carbon as well as carbon contamination (XPS spectra were measured before (A) and after (B) annealing).

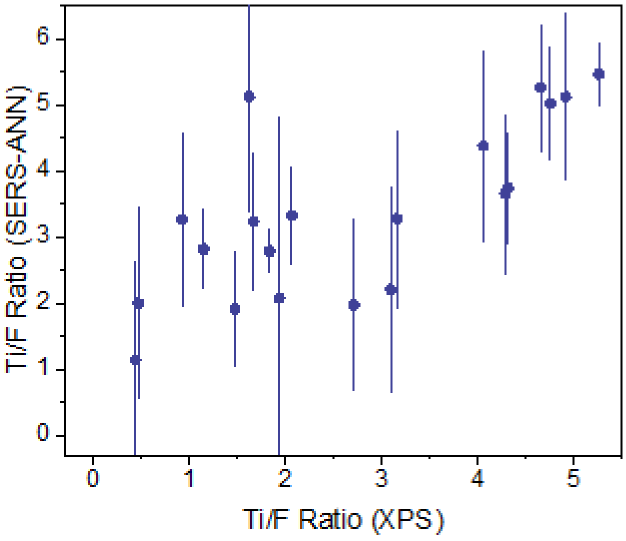

Figure S2 “Bad” results of $Ti_3C_2T_x$ flakes SERS-ANN based surface termination revealing (ANN training and SERS-ANN verification were performed including the data from a more simple, portable Raman spectrometer).

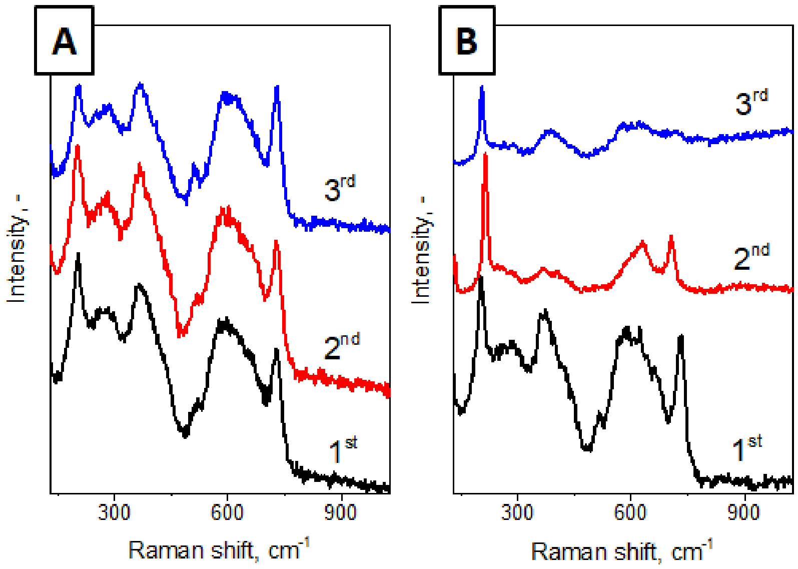

Figure S3 Demonstration of $Ti_3C_2T_x$ flakes degradation during the Raman measurements: SERS spectra measured three times consecutively in one and the same place, the case of stable $Ti_3C_2T_x$ flakes, prepared by one of the partners (A) and the case of “less-stable” flakes, prepared by another partner (B).

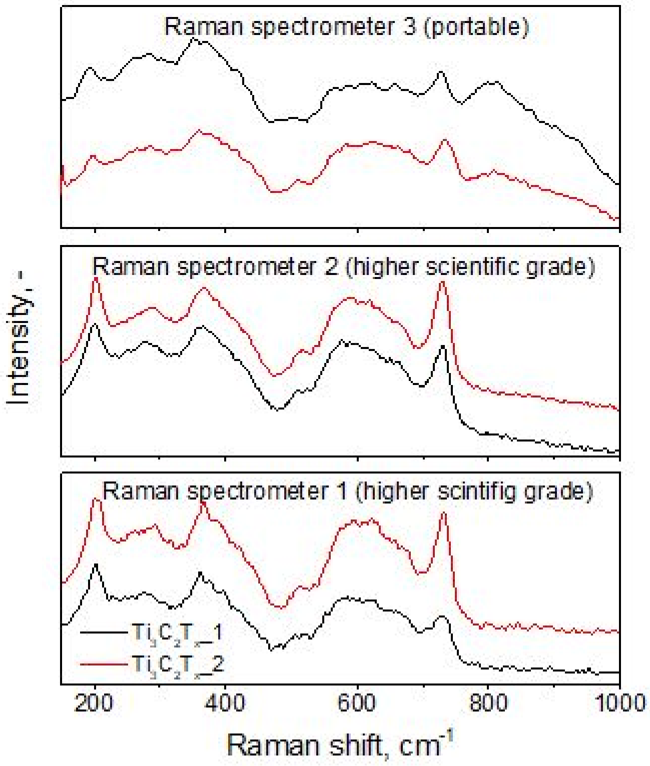

Figure S4 Comparison of SERS spectra, measured on three different Raman spectrometers.

3. ANN design and implementation

3.1 Data preprocessing

To standardize the data and allow using spectra from different devices for neural network training, SERS spectra were resampled to a standardized range. Linear interpolation was used for resampling and target range was selected as 100 – 1000 cm^{-1} with 1024 intermediate points in this interval. After resampling, the background was removed from the data using arPLS algorithm[1], and scale of each spectrum was normalized by subtracting the mean and dividing by the standard deviation. Spectra were split into 75:25 ration intro training and validation subsets.

3.2 Proposed architecture

The architecture of the neural network was chosen taking into account two main goals of data processing, i.e. (i) prediction of flakes surface composition, (ii) ability to simultaneously use data collected with different spectrometers, and (iii) analysis of differences in spectra from spectrometers or differences between flakes prepared by partners research teams. The second task shares many similarities with neural style transfer, i.e. a method which decomposes input data into the "meaningful" variability part and so-called style, which includes other data variability. One can imagine the handwritten dataset as an example: the style transfer method should decompose the data variability into class (which digit it is) and style (inclination, thickness, roundness, and other features of the handwriting). This task was effectively solved with conditional variational autoencoders (CVAE)[2], a neural network architecture able to disentangle the high-dimensional data manifold and "concentrate" the information into low dimensional space, where the position of the point encodes style of the input data. Thus, we took variation auto encoder as a basic architecture for our task, which however, required modifications to be able to solve the regression task.

Classical CVAE requires known data labels to perform style transfer, while the neural regression model is capable of predicting target variable, but is not able to perform style transfer. Here, we propose a regressive variational autoencoder (RegVAE), capable of doing both tasks simultaneously. The model is schematically shown in Fig. S4.

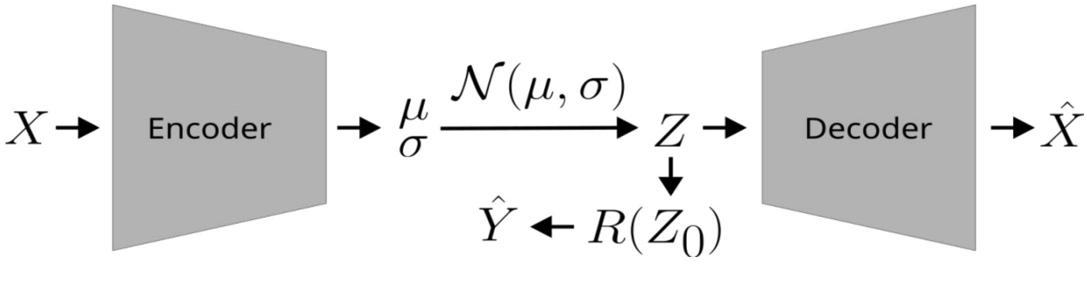

Figure S4 Schematic representation of RegVAE architecture.

In short, the model follows the structure of a classical variational autoencoder, consisting of the encoder, mapping the input vector **X** into distribution parameters **μ** and **σ**, and decoder sampling data **Z** from a normal distribution, defined by **μ** and **σ** and mapping it into reconstructed data **X̃**. We propose to treat one of the axes of **Z** as a regression predictions, which will be used to predict the target variable **Y**. For simplicity let us assume that the first axis, i.e. $\mathbf{Z}_0$ is used for regression. We use the value of $\mathbf{Z}_0$ directly because target Y can have a different scale / have a bias. Thus, we add one auxiliary linear neuron R, which scales $\mathbf{Z}_0$ to match **Y**. The network then is trained to minimize three losses simultaneously:

- - mean squared error between **X** and **X̃** (reconstruction loss, same as in classical autoencoder),
- - Kullback–Leibler divergence between Z and normal distribution (latent loss, same as in CVAE),
- - mean squared error between **Y** and R(**Z**[0]) (regression loss).

During training model learns mapping $\mathbf{X} \mapsto \mathbf{Z}$, such that the first dimension of **Z** is proportional to the regression target **Y**, while other dimensions define style. Due to the architecture of the variational autoencoder it is guaranteed that all dimensions of **Z** are not correlated, thus achieving disentanglement of regression variable and style information.

3.3 Implementation details

The model was implemented in the TensorFlow framework[3]. Encoder is built from 3 convolutional blocks, each block consisting of 1d convolution, layer normalization, activation, and max pooling with pool size 2. Blocks have 8, 16, and 4 filters of size 8, 16, and 1 respectively, GeLU activation[4] was used in all layers. The convolutional part is followed by the fully-connected part, consisting of three layers, containing 128, 96, and D*2 neurons, forming model output. D here is latent space dimensionality and was chosen to be equal to 6 from experiments (i.e. 1 regressive dimension and 5 style dimensions). The architecture of decoder mirrors the encoder architecture with max pooling operation replaced with upsampling.

Assignment of weights to losses allows us to find the balance between different objectives – quality of reconstruction, normality of latent space, and quality of regression. In our experiments, we found that assignment of weight 3 to both reconstruction and regression loss improved both regression quality and structure of the latent space. The model was trained for 200 epochs with Adam optimizer (with standard parameters) and a batch size of 32.

4. References

(1) Baek, S.-J.; Park, A.; Ahn, Y.-J.; Choo, J. Baseline Correction Using Asymmetrically Reweighted Penalized Least Squares Smoothing. *The Analyst* **2015**, *140* (1), 250–257. https://doi.org/10.1039/c4an01061b.

(2) Kingma, D. P.; Welling, M. An Introduction to Variational Autoencoders. *Found. Trends® Mach. Learn.* **2019**, *12* (4), 307–392. https://doi.org/10.1561/2200000056.

(3) Abadi, M.; Agarwal, A.; Barham, P.; Brevdo, E.; Chen, Z.; Citro, C.; Corrado, G. S.; Davis, A.; Dean, J.; Devin, M.; Ghemawat, S.; Goodfellow, I.; Harp, A.; Irving, G.; Isard, M.; Jia, Y.; Jozefowicz, R.; Kaiser, L.; Kudlur, M.; Levenberg, J.; Mane, D.; Monga, R.; Moore, S.; Murray, D.; Olah, C.; Schuster, M.; Shlens, J.; Steiner, B.; Sutskever, I.; Talwar, K.; Tucker, P.; Vanhoucke, V.; Vasudevan, V.; Viegas, F.; Vinyals, O.; Warden, P.; Wattenberg, M.; Wicke, M.; Yu, Y.; Zheng, X. TensorFlow: Large-Scale Machine Learning on Heterogeneous Distributed Systems. arXiv March 16, 2016. https://doi.org/10.48550/arXiv.1603.04467.

(4) Hendrycks, D.; Gimpel, K. Gaussian Error Linear Units (GELUs). arXiv June 5, 2023. https://doi.org/10.48550/arXiv.1606.08415.
